# Untapped Potential of Artificial Intelligence for Analysis of Epileptic Seizure Videos: A Clinician’s Expectation

**DOI:** 10.1016/j.mcpdig.2024.01.004

**Published:** 2024-02-15

**Authors:** Naotaka Usui

**Affiliations:** NHO Shizuoka Institute of Epilepsy and Neurological Disorders, Shizuoka, Japan

Epilepsy is a very common neurological disorder, affecting 1%-2 % of the population worldwide. It is characterized by recurrent epileptic seizures which are defined as transient occurrence of signs or symptoms due to abnormal excessive or synchronous neuronal activities in the brain. In the field of epilepsy, artificial intelligence (AI) has been tested for the classification of epilepsy, electroencephalogram (EEG) analysis, and magnetic resonance imaging detection of epileptogenic lesions such as focal cortical dysplasia, etc. However, there are a relatively small number of papers regarding AI for seizure semiology.

Analysis of seizure semiology is crucial for the proper diagnosis of epilepsy. It is important to discriminate true epileptic seizures from nonepileptic events such as syncope or psychogenic nonepileptic seizures because misdiagnosis may lead to inadequate treatment and adverse events. It is also important to differentiate different types of epileptic seizures, such as focal seizures and generalized seizures for appropriate use of antiseizure medications. In about one-third of epilepsy patients, seizures are medically intractable. In these patients, epilepsy surgery should be considered. In the presurgical evaluation of epilepsy, comprehensive examination including video-EEG monitoring, neuroimaging such as magnetic resonance imaging and functional neuroimaging, and neuropsychological testing is important for the estimation of the epileptogenic zone (EZ) that should be surgically resected for seizure relief. Correct diagnosis of EZ leads to seizure freedom after epilepsy surgery. Analysis of seizure semiology plays a pivotal role also in the presurgical evaluation of epilepsy. Epileptic seizures contain variable semiology including subjective symptoms such as sensory, mnemonic or affective domains, altered awareness, motor phenomena such as automatisms and convulsive movements, and autonomic phenomena. Video-recorded objective symptoms are visually analyzed by experienced epileptologists. Seizure symptoms sometimes provide clues for lateralization of EZ. Usually, only 1 symptom is not enough for localizing EZ. A combination and sequence of symptoms may provide localizing information on EZ. Facial symptoms such as color, oral movements, bilateral tonic facial contraction, and emotional expression provide rich information and are important for correct diagnosis of EZ. Facial emotional expressions such as fear, disgust, mirth, etc. provide important clues for the localization of seizure origin. The correct interpretation of semiology may be more difficult than the analysis of EEG and neuroimaging and requires expertise.

To improve the general level of epilepsy diagnosis and treatment, education of seizure semiology for physicians is of paramount importance. Recorded seizure videos of patients are indispensable for education, however, at the same time, privacy of patients must be protected. The eyes of patients are commonly masked for deidentification for presentation at conferences or publications. However, many seizure symptoms such as eye deviation have been lost by masking of eyes. Therefore, the development of methodology preserving the complex seizure semiology, while protecting the privacy of the patients is strongly desired. In this context, McGonigal et al^1^ tested the feasibility of applying facial deidentification to seizure videos, degree of deidentification achieved with different models, and the preservation of rich facial semiologic information. They used an AI-based face-swapping approach, which was already tested in movement disorder research but has not been tested in the field of epilepsy yet. As the authors wrote in the paper, reliable means of deidentifying facial video data in medical videos could help mitigate privacy concerns, allowing for more effective data sharing that would be useful for research, teaching, and training purposes. They used a single representation video frame prepared per seizure, and tested 3 AI transformation models, (1) morphing the original face image with a different male face; (2) substitution with a female face; and (3) cartoonization. Facial deidentification and preservation of clinically relevant facial details were calculated based on scoring by 5 independent expert clinicians, and objective computation. The results showed that the best compromise between deidentification and preservation of facial semiology was cartoonization. This study is a valuable first attempt using AI methods of transformation of seizure videos for deidentification and preservation of important semiology. If the methodology is further improved, it will be very useful for data sharing and WEB conferences between epilepsy centers, and for other educational purposes, and their efforts will lead to further improvement of epilepsy care. The strength of their paper is the use of both evaluation by experienced clinicians and by objective computational scores.

The main limitation is that they analyzed a single video frame, not dynamic videos, although it is quite understandable that they started their research using a single video frame. Seizure semiology such as automatisms, and facial expressions can be more correctly analyzed by dynamic videos. Single video frames are not suitable for analyzing such seizure semiology precisely. Facial semiology, especially facial expression is very intricate, and the interpretation sometimes depends on the interpreter. Although some simple motor phenomena such as asymmetrical tonic posturing are identifiable with a single video frame, emotional facial features are difficult to interpret. The emotional contents of the figure provided on the paper seem to be different between the original face and the transformed faces. A single video frame will be useful for education of certain symptoms such as asymmetric tonic limb posturing. However, it may be still premature to use a transformed single video image presented for correct semiologic diagnosis, especially for emotional facial expressions. For analyzing and educating facial symptoms and motor symptoms precisely, dynamic videos are indispensable. In the next step, the authors will proceed to the use of dynamic videos, although it may need more computational power.

Because the visual interpretation of semiology depends largely on the subjectivity of experienced epileptologists, automated methods for seizure detection and classification have been also attempted. Ahmedt-Aristizabal et al^2^ reported a deep learning approach for classifying mesial temporal lobe epilepsy and extratemporal epilepsy relying on the fusion of facial expression and pose dynamics. Moro et al^3^ reported their automatic method using a deep learning approach based on video analysis for the characterization of human motion in patients with sleep-related hypermotor epilepsy. Their pipeline successfully discriminated different sleep-related hypermotor epilepsy semiology patterns and disorders of arousal with a test accuracy of 80%. These methodologies may provide quantitative semiologic information, which is not discernable by visual analysis alone, and provide objective analysis for clinicians. It is expected that these methods will improve the analysis of seizure semiology and support clinical decision-making in the near future. At the same time, the research of seizure semiology itself must be developed further.

The application of AI for epilepsy, especially for seizure semiology is still underdeveloped. In this regard, the work by McGonigal et al^1^ is very stimulating. For the development of AI in the field of epilepsy, development of both AI and clinical epileptology would be necessary. Collaboration between clinicians and AI technologists is essential. The AI analysis will provide further insight into our deep understanding of seizure semiology.

## Potential Competing Interests

The authors report no competing interest.
